# Immunological correlates of protection mediated by a whole organism, *Cryptococcus neoformans*, vaccine deficient in chitosan

**DOI:** 10.1128/mbio.01746-24

**Published:** 2024-07-09

**Authors:** Charles A. Specht, Ruiying Wang, Lorena V. N. Oliveira, Maureen M. Hester, Christina Gomez, Zhongming Mou, Diana Carlson, Chrono K. Lee, Camaron R. Hole, Woei C. Lam, Rajendra Upadhya, Jennifer K. Lodge, Stuart M. Levitz

**Affiliations:** 1 Department of Medicine, University of Massachusetts Chan Medical School, Worcester, Massachusetts, USA; 2 Department of Molecular Microbiology, Washington University School of Medicine, St. Louis, Missouri, USA; University of Minnesota Medical School, Minneapolis, Minnesota, USA

**Keywords:** live vector vaccines, mycology, *Cryptococcus neoformans*, T cells, AIDS

## Abstract

The global burden of infections due to the pathogenic fungus *Cryptococcus* is substantial in persons with low CD4^+^ T-cell counts. Previously, we deleted three chitin deacetylase genes from *Cryptococcus neoformans* to create a chitosan-deficient, avirulent strain, designated as *cda1∆2∆3∆*, which, when used as a vaccine, protected mice from challenge with virulent *C. neoformans* strain KN99. Here, we explored the immunological basis for protection. Vaccine-mediated protection was maintained in mice lacking B cells or CD8^+^ T cells. In contrast, protection was lost in mice lacking α/β T cells or CD4^+^ T cells. Moreover, CD4^+^ T cells from vaccinated mice conferred protection upon adoptive transfer to naive mice. Importantly, while monoclonal antibody-mediated depletion of CD4^+^ T cells just prior to vaccination resulted in complete loss of protection, significant protection was retained in mice depleted of CD4^+^ T cells after vaccination but prior to challenge. Vaccine-mediated protection was lost in mice genetically deficient in interferon-γ (IFNγ), tumor necrosis factor alpha (TNFα), or interleukin (IL)-23p19. A robust influx of leukocytes and IFNγ- and TNFα-expressing CD4^+^ T cells was seen in the lungs of vaccinated and challenged mice. Finally, a higher level of IFNγ production by lung cells stimulated ex vivo correlated with lower fungal burden in the lungs. Thus, while B cells and CD8^+^ T cells are dispensable, IFNγ and CD4^+^ T cells have overlapping roles in generating protective immunity prior to *cda1∆2∆3∆* vaccination. However, once vaccinated, protection becomes less dependent on CD4^+^ T cells, suggesting a strategy for vaccinating HIV^+^ persons prior to loss of CD4^+^ T cells.

**IMPORTANCE:**

The fungus *Cryptococcus neoformans* is responsible for >100,000 deaths annually, mostly in persons with impaired CD4^+^ T-cell function such as AIDS. There are no approved human vaccines. We previously created a genetically engineered avirulent strain of *C. neoformans*, designated as *cda1∆2∆3∆*. When used as a vaccine, *cda1∆2∆3∆* protects mice against a subsequent challenge with a virulent *C. neoformans* strain. Here, we defined components of the immune system responsible for vaccine-mediated protection. We found that while B cells and CD8^+^ T cells were dispensible, protection was lost in mice genetically deficient in CD4^+^ T cells and the cytokines IFNγ, TNFα, or IL-23. A robust influx of cytokine-producing CD4^+^ T cells was seen in the lungs of vaccinated mice following infection. Importantly, protection was retained in mice depleted of CD4^+^ T cells following vaccination, suggesting a strategy to protect persons who are at risk of future CD4^+^ T-cell dysfunction.

## INTRODUCTION

Cryptococcosis, due to the encapsulated species of fungi including *Cryptococcus neoformans* and *Cryptococcus gattii*, is a major cause of morbidity and mortality worldwide. Human exposure is thought to mainly occur following inhalation of airborne organisms. A pulmonary infection may result, which is often asymptomatic. In the absence of effective host defenses, infection can spread locally and also disseminate, most often to the central nervous system, where it causes meningoencephalitis. Most persons with cryptococcosis have quantitative or qualitative CD4^+^ T-cell dysfunction. The estimated global burden of cryptococcosis is 194,000 incident cases per year with 147,000 deaths (1). An estimated 19% of AIDS-related deaths are due to cryptococcal meningitis (2). Other immunosuppressed persons are at high risk; e.g., 1%–5% of solid organ transplant recipients develop cryptococcosis (3). *C. neoformans* is found worldwide, while *C. gattii* is endemic to tropical and subtropical regions. In addition, hypervirulent strains of *C. gattii* have emerged in northwestern regions of North America, most notably on Vancouver Island (4–6).

Despite the need, there are no vaccines to protect humans from cryptococcosis (7). Populations which could be targeted for vaccination include persons who are (i) HIV infected, (ii) on medications which suppress T cells (particularly transplant recipients), (iii) living in *C. gattii*-endemic regions, and (iv) with other high-risk diseases (e.g., sarcoidosis and lymphoma). Protection against experimental cryptococcosis can be obtained by immunization with cryptococcal strains missing virulence factors (such as chitosan, capsule, sterylglucosidase, and the F-box protein, Fbp1) or engineered to heterologously express interferon-γ (IFNγ) or overexpress the transcription factor Znf2 (8–15). In addition, protection can be obtained by immunizing mice with recombinant protein subunit vaccines adjuvanted in glucan particles (16, 17).

Previously, we deleted three chitin deacetylase genes from the highly virulent KN99 strain of *C. neoformans*; the resulting strain, designated as *cda1∆2∆3∆*, is unable to produce chitosan, the deacetylated form of chitin, in the cryptococcal cell wall (11, 18). The *cda1∆2∆3∆* strain is rapidly cleared from the lungs of CBA/J mice (11, 19). Mice that are given a pulmonary vaccination with live or heat-killed (HK) *cda1∆2∆3∆* yeast cells are protected from a subsequent lethal lung challenge with the *C. neoformans* strain KN99 and (albeit to a lesser extent) *C. gattii* strain R265 (11, 20). Wild-type (WT) mice typically die within 4 weeks following infection with the virulent *C. neoformans* KN99 strain, with dissemination to the brain as seen in most lethal human infections. In contrast, survival of vaccinated mice typically approaches 100%. In the present study, we explored the immunological basis for *cda1∆2∆3∆* vaccine-mediated protection against pulmonary challenge with the *C. neoformans* KN99 strain.

## RESULTS

### The kinetics of pulmonary clearance of live *cda1∆2∆3∆* in CBA/J, BALB/c, C57BL/6, and NSG mice

**Fig 1:**
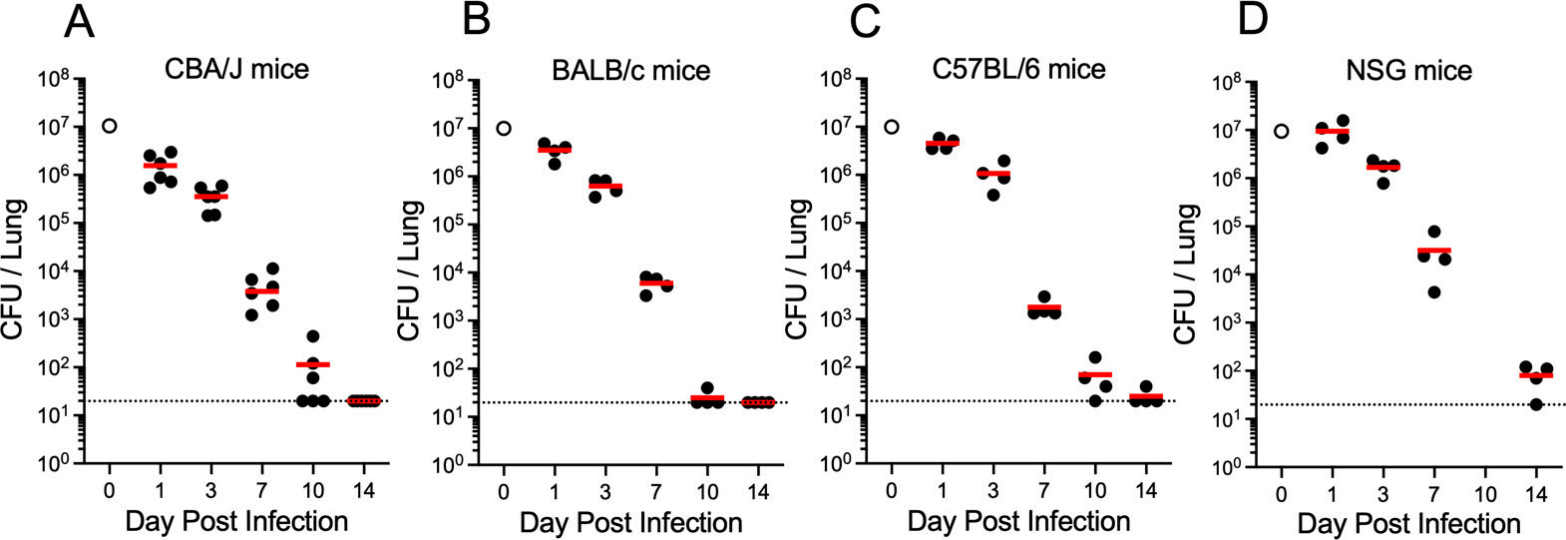
The kinetics of pulmonary clearance of *cda1∆2∆3∆* in multiple mouse strains. CBA/J (**A**), BALB/c (**B**), C57BL/6 (**C**), and NSG (**D**) mice were infected orotracheally with 1 × 10^7^ CFU of live *cda1∆2∆3∆* strain. At designated days post infection, CFUs in the lungs were determined. Infected mice had *n* = 4–6 mice per time point. Each circle represents CFUs in an individual mouse. Red horizontal bars denote mean values. Dotted lines at 20 CFU indicate the detection limit for CFU quantification. The inoculum is represented by the open circle at day 0.

We previously reported that the *cda1∆2∆3∆* strain was rapidly cleared from the lungs of wild-type CBA/J mice (11, 19). In the first set of experiments, we extended the studies to determine the clearance rate of *cda1∆2∆3∆* at a vaccination dose of 1 × 10^7^ colony-forming unit (CFU) for CBA/J, BALB/c, C57BL/6, and non-obese diabetic, severe combined immunodeficiency, IL-2R common gamma-chain deficient (NSG) mice. The NSG mouse strain has particular translational relevance for use of live vaccines in immunocompromised populations, as it is important to demonstrate the vaccine strain is also avirulent in severely immunodeficient mice. NSG mice have multiple mutations, making them highly immunodeficient. They lack mature B cells, T cells, and natural killer cells, and have defects in innate immunity and signaling pathways mediated by cytokine receptors which share the gamma chain (21). When CBA/J, BALB/c, C57BL/6, and NSG mice were infected with 1 × 10^7^ *cda1∆2∆3∆* yeast cells, CFUs in mouse lungs declined in a logarithmic manner to near undetectable levels over the 14-day time course of the experiment (Fig. 1).

### Role of B cells and antibody in protection mediated by the *cda1∆2∆3∆* vaccine

Having demonstrated that *cda1∆2∆3∆* cells were avirulent in NSG mice that lacked B and T cells, we next systematically examined the contribution of B cells, CD8^+^ T cells, and CD4^+^ T cells to protection against cryptococcosis mediated by the live *cda1∆2∆3∆* vaccine. The protective efficacy of the *cda1∆2∆3∆* vaccine against pulmonary challenge with *C. neoformans* was examined in two strains of mice congenitally deficient in B cells. JHD mice (BALB/c background) lack mature B cells due to a targeted deletion of JH gene segments and are completely devoid of immunoglobulins (22). muMT mice (C57BL/6 background) lack B cells due to targeted disruption of the mu heavy chain gene (23); they make immunoglobulins at levels that are >100-fold lower than in normal mice (24).

**Fig 2:**
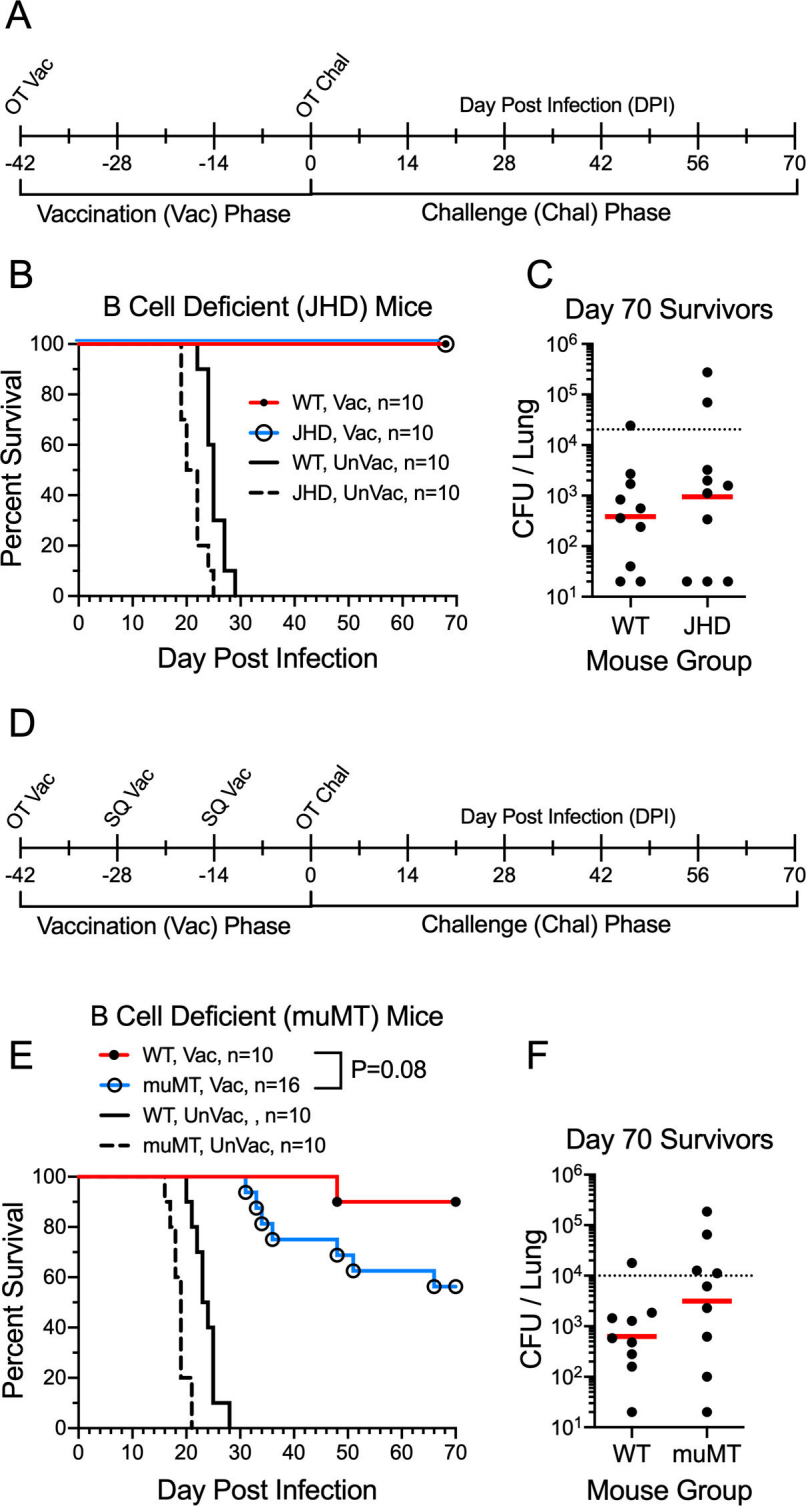
Contribution of B cells and antibody to protection following *cda1∆2∆3∆* vaccination. (**A**) Protocol for experiments with wild-type (WT) BALB/c and JHD mice. Mice were given orotracheal (OT) vaccination with 1 × 10^7^ CFU live *cda1∆2∆3∆* and challenge with 2 × 10^4^ CFU KN99. (**B**) Survival rates of vaccinated (Vac) and unvaccinated (UnVac) WT BALB/c and B cell-deficient JHD mice were compared. (**C**) CFUs in the lungs of mice which survived to 70 DPI were determined. (**D**) Protocol for experiments with WT C57BL/6 and muMT mice as in panel A, except the mice were also given two subcutaneous boosts of 2 × 10^6^ CFU live *cda1∆2∆3∆*. (**E**) Survival rates of Vac and UnVac wild-type C57BL/6 and muMT mice were compared. (**F**) CFUs in the lungs of mice which survived to 70 DPI were determined. Kaplan-Meier survival curves were compared using the Mantel-Cox log-rank test. For each strain of mice, *P* < 0.001 comparing vaccinated with unvaccinated mice. Data are from ≥2 independent experiments. The number (*n*) of mice per group is indicated in the figure inset. For CFU, each circle represents the CFU of an individual mouse. Red horizontal bars denote geometric mean values. Dotted lines indicate the KN99 challenge dose.

Wild-type BALB/c and B cell-deficient JHD mice were vaccinated and then given a pulmonary challenge 42 days later with the virulent KN99 strain of *C. neoformans*, after which they were monitored for survival (Fig. 2A). Survival of both mouse strains was 100% when the experiment was terminated 70 days post infection (DPI) (Fig. 2B). Moreover, when the experiment was terminated on day 70, lung CFUs were similar, comparing WT and JHD mice (Fig. 2C). Similar experiments were performed in wild-type C57BL/6 and muMT mice, except the mice were given three vaccinations [orotracheal (OT) followed by two biweekly subcutaneous boosts] prior to infection (Fig. 2D). The vaccine boosts were given because while a single vaccine dose confers significant protection to C57BL/6 mice, the protection is not as robust compared with other mouse strains (11). Wild-type C57BL/6 and muMT mice were protected by vaccination, although protection was less than 100% (Fig. 2E). There was a trend towards decreased survival of the muMT mice compared to that seen in similarly vaccinated wild-type C57BL/6 mice. Lung CFUs in surviving mice did not significantly differ when comparing wild-type C57BL/6 and muMT mice. All unvaccinated mice, regardless of the mouse strain, succumbed within 30 DPI (Fig. 2B and E).

### Role of α/β T cells and CD8^+^ T cells in protection mediated by the *cda1∆2∆3∆* vaccine

**Fig 3:**
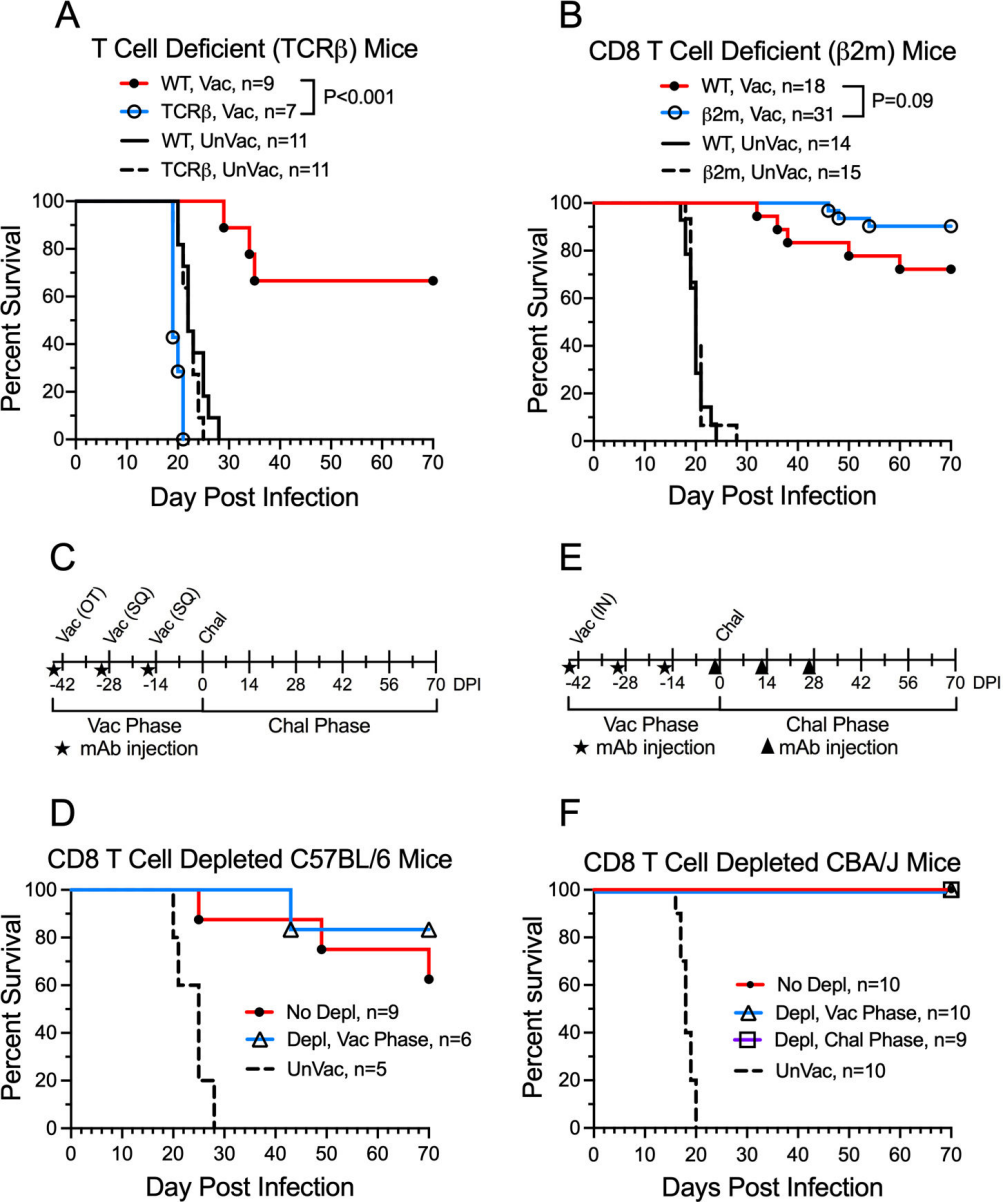
Contribution of CD8^+^ T cells to protection mediated by vaccination with *cda1∆2∆3∆*. (**A**) Wild-type (WT) C57BL/6 mice and α/β T cell-deficient [T-cell receptor beta (TCRβ)] mutant mice on the C57BL/6 background were given an OT vaccination (1 × 10^7^ CFU) and two biweekly subcutaneous (SQ) boosts (2 × 10^6^ CFU each) with *cda1∆2∆3∆*. Two weeks after the second SQ boost, mice were challenged with 1 × 10^4^ CFU KN99 and followed for 70 days for survival. (**B**) As in panel A except CD8^+^ T cell-deficient (β2m) mice were compared to WT mice. (**C**) Protocol for vaccinations with *cda1∆2∆3∆* and intraperitoneal injections of CD8^+^ T cell-depleting mAb 2.43 to C57BL/6 mice during the vaccination (Vac) phase. Mice were challenged (Chal) with 1 × 10^4^ CFU KN99 and followed for 70 days for survival. (**D**) Survival curves of C57BL/6 mice treated according to the protocol in panel C. (**E**) As in panel C except the CD8^+^ T cell-depleting mAb YTS 169.4 was administered biweekly to CBA/J mice during both the Vac and Chal phases, and the inoculum was 5 × 10^4^ CFU KN99. (**F**) Survival curves of CBA/J mice treated according to the protocol in panel E. Statistics by Mantel-Cox log rank test. Data are from ≥2 independent experiments. The number (*n*) of mice per group is indicated in the figure inset. UnVac, unvaccinated. Depl, depleted.

We next turned our attention to the role of T cells. T-cell receptor beta (TCRβ)-deficient mice (C57BL/6 background) contain a targeted deletion of T-cell receptor β-chain and lack α/β T cells, but have normal differentiation of γ/δ T cells (25). The *cda1∆2∆3∆* vaccine failed to protect TCRβ mice from a lethal challenge with *C. neoformans* (Fig. 3A), strongly suggesting a critical role for α/β T cells in vaccine-mediated protection. As TCRβ mice lack both CD4^+^ and CD8^+^ α/β T cells, we next examined whether the CD8^+^ T-cell subset contributed to protection by comparing survival following cryptococcal challenge of vaccinated wild-type C57BL/6 and β-microglobulin knockout (β2m) mice. β2m mice lack surface expression of major histocompatibility complex (MHC) class I and are virtually devoid of CD8^+^ T cells (26). The survival curves did not significantly differ; in fact, there was a non-significant trend toward increased survival in the β2m mice (Fig. 3B).

β2m mice have perturbations aside from CD8 deficiency, including elevated iron levels, reduced neonatal Fc receptor function, and deficient natural killer T-cell activity (27–29). As an alternative method to examine the role of CD8^+^ T cells, we treated mice with monoclonal antibodies (mAbs) 2.43 and YTS 169.4 to deplete CD8^+^ T cells from C57BL/6 mice and CBA/J mice, respectively (30). This also allowed us to expand studies on the importance of CD8^+^ T cells for protection to a second strain of mice, CBA/J. Confirmation that these mAbs were effective at depleting CD8^+^ T cells in C57BL/6 and CBA/J mice was shown by fluorescence-activated cell sorting (FACS) (Fig. S1A and B). For the studies with C57BL/6 mice, mAb 2.43 was administered 2 days prior to each of the three vaccinations, and then the mice were followed for survival following fungal challenge (Fig. 3C and D). For the studies with CBA/J mice, three biweekly doses of mAb YTS 169.4 were administered either during the vaccine phase or during the challenge phase of the experiment (Fig. 3E and F). Regardless of when CD8^+^ T-cell depletion was performed, vaccinated C57BL/6 and CBA/J mice had >80% survival following the *C. neoformans* challenge (Fig. 3D and F). Moreover, survival was not significantly different comparing mAb-treated mice and mice with intact CD8^+^ T-cell populations.

### Role of CD4^+^ T cells in protection mediated by the *cda1∆2∆3∆* vaccine

**Fig 4:**
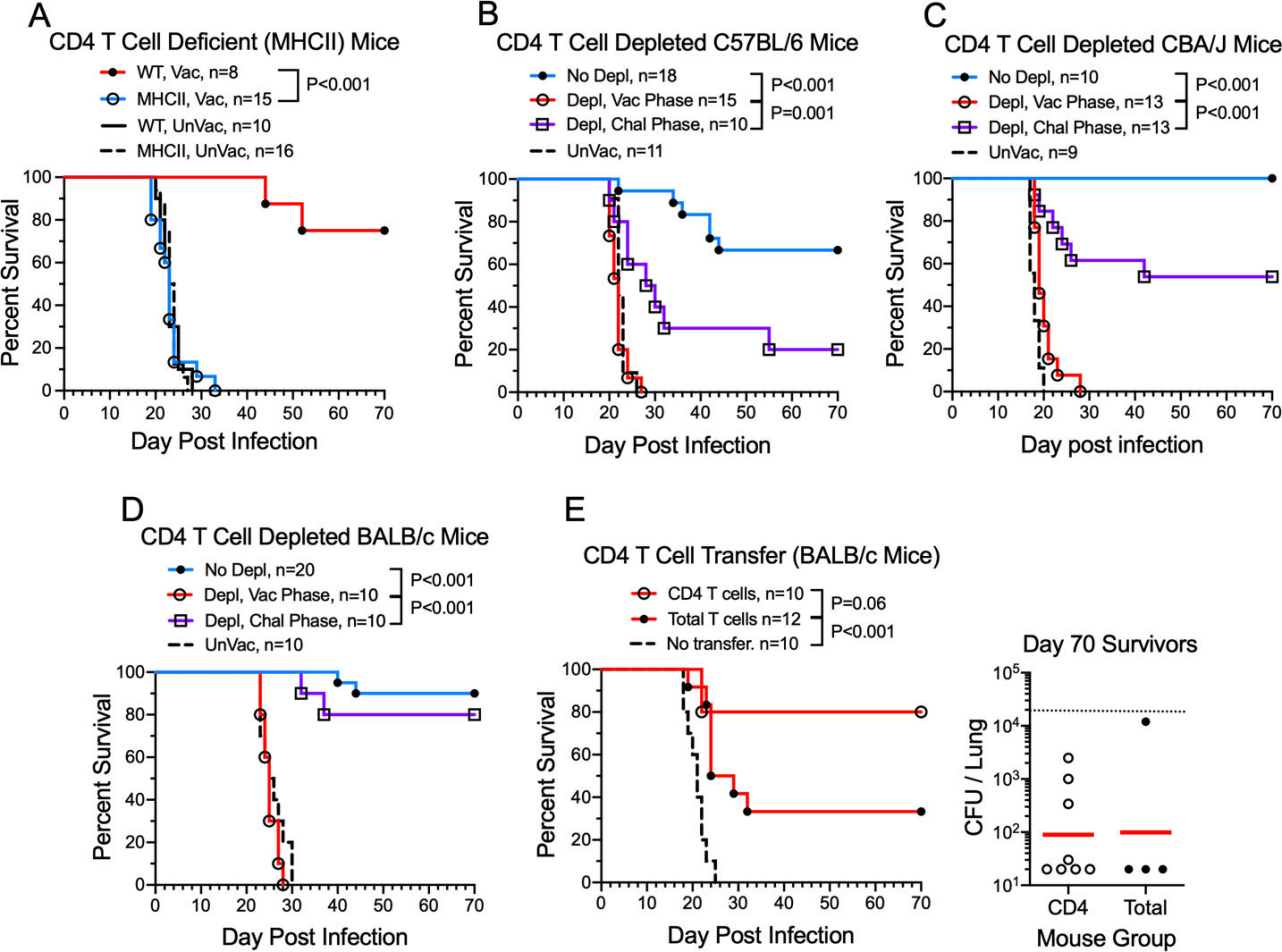
Contribution of CD4^+^ T cells to protection afforded by *cda1∆2∆3∆* vaccination. (**A**) Vaccinated wild-type C57BL/6 (WT) and CD4^+^ T cell-deficient (MHCII) mice were given three biweekly vaccinations (OT followed by two SQ boosts) with *cda1∆2∆3∆*. Two weeks after the last boost, mice were challenged with 1 × 10^4^ CFU KN99. (**B–D**) Survival of vaccinated C57BL/6 (**B**), CBA/J (**C**), and BALB/c (**D**) mice following three biweekly injections of CD4^+^ T cell-depleting mAb GK1.5 during the vaccinated (Depl, Vac) phase or challenged (Depl, Chal) phase. (**E**) Intravenous transfer of total T cells or CD4^+^ T cells purified from spleens of BALB/c mice vaccinated with *cda1∆2∆3∆* to naïve BALB/c mice followed by challenge with KN99. At 70 DPI, surviving mice were euthanized, and lung CFUs of each mouse were determined. For CFU, each circle represents the CFU of an individual mouse. Red horizontal bars denote geometric mean values. The dotted line at 2 × 10^4^ indicates the KN99 challenge dose. Statistics by Mantel-Cox log rank test. Data are from ≥2 independent experiments. The number (*n*) of mice per group is indicated in the figure inset. UnVac, unvaccinated.

The lack of a phenotype with mice lacking CD8^+^ T cells suggested CD4^+^ T cells were critical for *cda1∆2∆3∆* vaccine-mediated protection. Therefore, we next examined vaccine-mediated protection in mice deficient in MHC class II [major histocompatibility locus class II (MHCII)]; these mice lack CD4^+^ T cells. MHCII mice vaccinated with *cda1∆2∆3∆* were not protected against lethal challenge with *C. neoformans*; the survival curves of vaccinated and unvaccinated MHCII mice were practically superimposable (Fig. 4A). To validate the observations about the importance of CD4^+^ T cells and to extend these observations to other mouse strains, we used the anti-CD4 mAb GK1.5 to deplete CD4^+^ T cells. Published data by our group and others demonstrated GK1.5 depletes the blood CD4^+^ T-cell population within 24 h of injection (31, 32). Moreover, depletion is sustained for at least 2 weeks, followed by a slow rebound in numbers (Fig. S1C and D). Three mouse strains, C57BL/6 (Fig. 4B), CBA/J (Fig. 4C), and BALB/c (Fig. 4D), were studied. The experimental scheme was similar to what was used to deplete CD8^+^ T cells in the experiments depicted in Fig. 3, except CBA/J and BALB/c mice were given an intranasal or OT vaccination but no subsequent subcutaneous (SQ) vaccinations. Mice were given three injections of GK1.5 at 2-week intervals during either the vaccination or challenge phase of the experiment. For each of the mouse strains, protection was lost when GK1.5 was administered during the vaccination phase. Importantly though, significant protection was retained if CD4^+^ T cells were depleted in the challenge phase. In BALB/c, the protection was similar to the no depletion control, while in CBA/J and C57BL/6, the protection was lessened but still significantly different from depletion in the vaccination phase. These data indicate that CD4^+^ T cells are essential for protection prior to vaccination but are not absolutely required to sustain the protection.

Finally, we performed adoptive transfer experiments to further assess the role of CD4^+^ T cells in vaccine-mediated protection (Fig. 4E). Splenocytes were prepared from BALB/c mice vaccinated with the *cda1∆2∆3∆* vaccine. Two groups of cells were purified, one consisting of total (CD3^+^) T cells and the other of the CD4^+^ T-cell subpopulation. Adoptive intravenous transfer of each group of cells protected naïve mice against *C. neoformans* challenge, with the protection afforded by the purified CD4^+^ T-cell population being more robust. Surviving mice showed similar clearance of KN99 from the lungs.

### The contribution of selected cytokines to *cda1∆2∆3∆* vaccine-mediated protection

**Fig 5:**
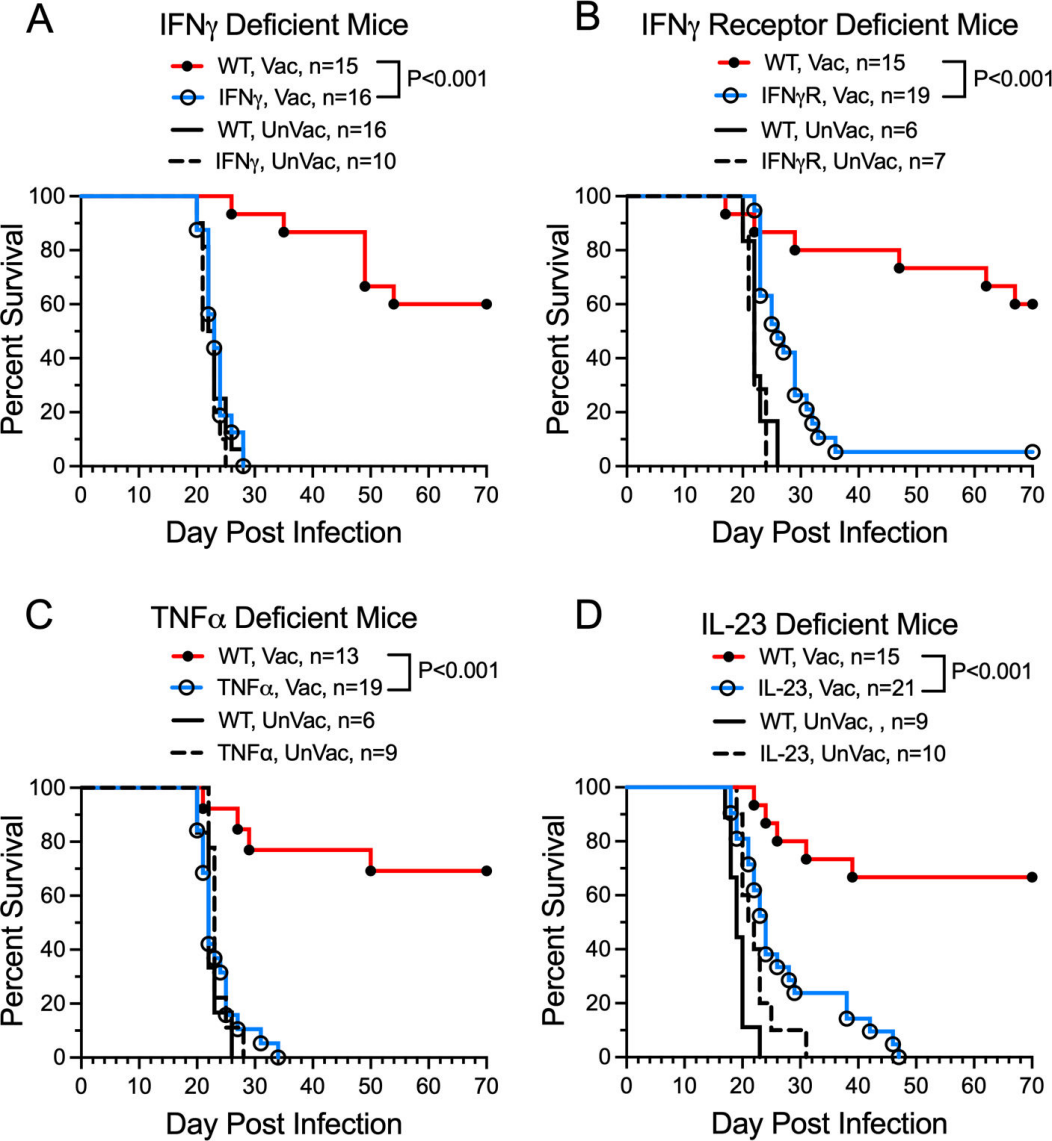
Contribution of selected cytokines to *cda1∆2∆3∆* vaccine-mediated protection. Survival rates of vaccinated (Vac) and unvaccinated (UnVac) wild-type (WT) C57BL/6 mice were compared with that of mice deficient in (**A**) IFNγ, (**B**) IFNγR, (**C**) TNFα, and (**D**) IL-23p19 following an OT challenge with KN99. Statistics by Mantel-Cox log rank test. Data are from ≥2 independent experiments. The number (*n*) of mice per group is indicated in the figure inset.

The finding that CD4^+^ T cells were critical to protection prompted us to explore the role of cytokines instrumental to their biasing and function. We focused on IFNγ, tumor necrosis factor alpha (TNFα), and interleukin (IL)-23 as these three cytokines have known benefit in murine models of cryptococcosis (32–36). Vaccine-mediated protection was completely, or nearly completely, abrogated in mice lacking IFNγ (Fig. 5A), IFNγR (Fig. 5B), TNFα (Fig. 5C), and IL-23p19 (Fig. 5D).

### The nature of the lung T-cell response following *cda1∆2∆3∆* vaccination and infection

The above experiments established that CD4^+^ T cells and the cytokines IFNγ, TNFα, and IL-23 were required for vaccine-mediated infection. We next examined the quantity and quality of the pulmonary immune response following vaccination and/or infection. BALB/c mice were vaccinated with an OT dose of live *cda1∆2∆3∆* vaccine and were challenged 6 weeks later with *C. neoformans* KN99. Control mice were left unvaccinated and/or uninfected. Mice were euthanized either before challenge (0 DPI) or post challenge (10 or 70 DPI), at which time their blood was collected by cardiac puncture, following which their lungs were harvested. As unvaccinated mice all die by 30 DPI, the 70-DPI group was composed of only vaccinated mice.

**Fig 6:**
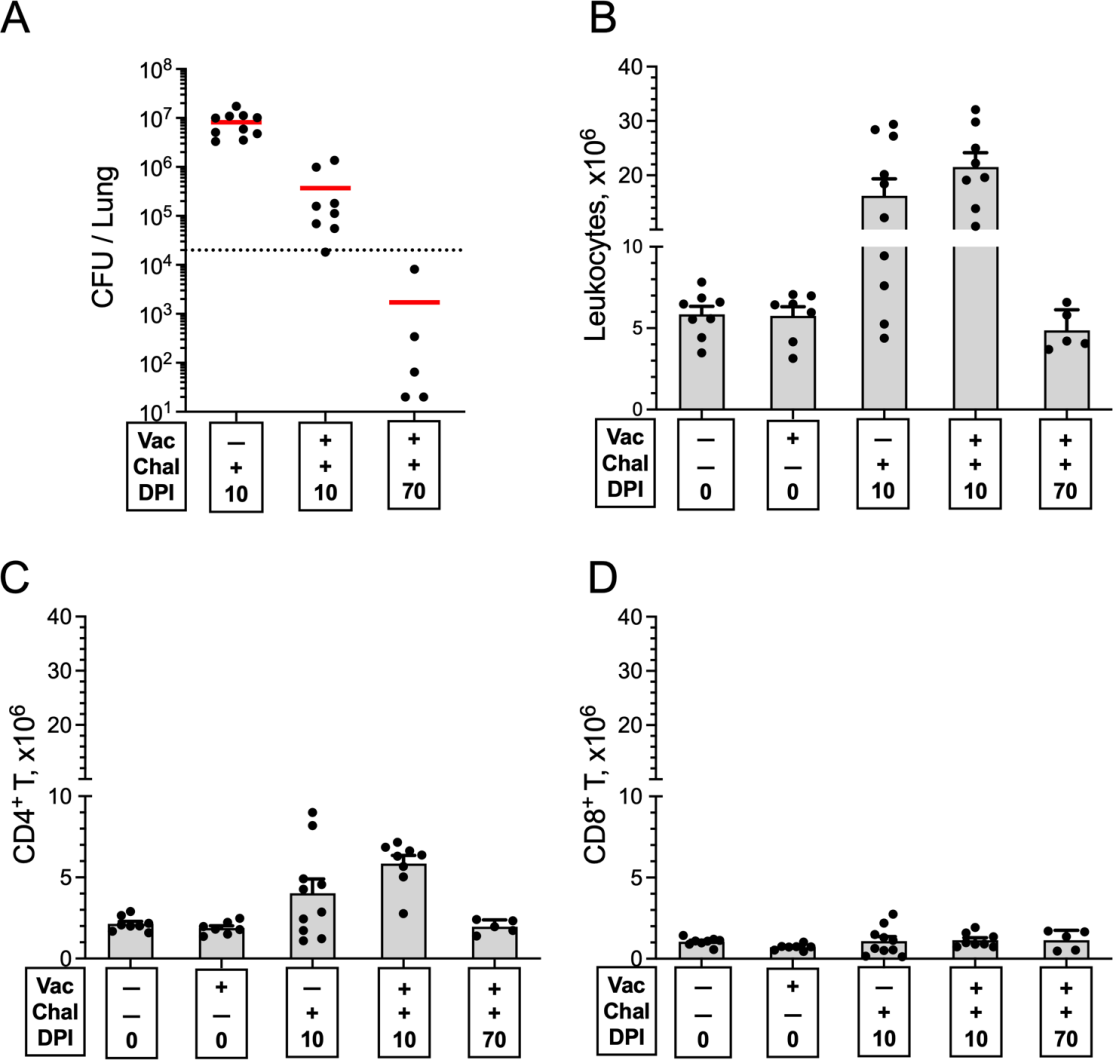
Quantitation of lung CFUs, leukocytes, and CD4^+^ and CD8^+^ T cells following vaccination and/or infection. BALB/c mice were vaccinated orotracheally with live *cda1∆2∆3∆*. Six weeks later, the mice were given a pulmonary challenge with KN99. Mice were euthanized at 0 (uninfected), 10, or 70 DPI. Controls included unvaccinated mice euthanized at 0 or 10 DPI. Lungs were harvested and single-cell suspensions were prepared. (**A**) CFUs per lung were determined. The horizontal bar represents the median CFUs. The dotted line depicts the challenge inoculum. (**B**) Leukocytes were purified on a Percoll gradient and counted. (**C and D**) The numbers of CD4^+^ and CD8^+^ T cells were calculated by multiplying the percentage of each population, as determined by FACS, times the total leukocyte count. Data are from two independent experiments, each with four to six mice/group (except for the 70-DPI group, which had two to three mice/group). The data are presented as mean ± SEM. Statistical comparisons between groups are shown in Table S1. Chal, challenged with *C. neoformans* KN99; DPI, days post infection; Vac, vaccinated.

Compared to unvaccinated mice at 10 DPI, vaccinated mice had significantly fewer CFUs in the lungs at 10 and 70 DPI (Fig. 6A). However, total numbers of lung leukocytes, CD4^+^ T cells, and CD8^+^ T cells were not significantly different comparing unvaccinated and vaccinated mice at the 0- and 10-DPI time points (Fig. 6B through D). Notably, numbers of total leukocytes and CD4^+^ T cells increased in infected mice at 10 DPI and then returned to near baseline levels at 70 DPI. In contrast, CD8^+^ T-cell counts did not significantly differ regardless of vaccination or infection status.

**Fig 7:**
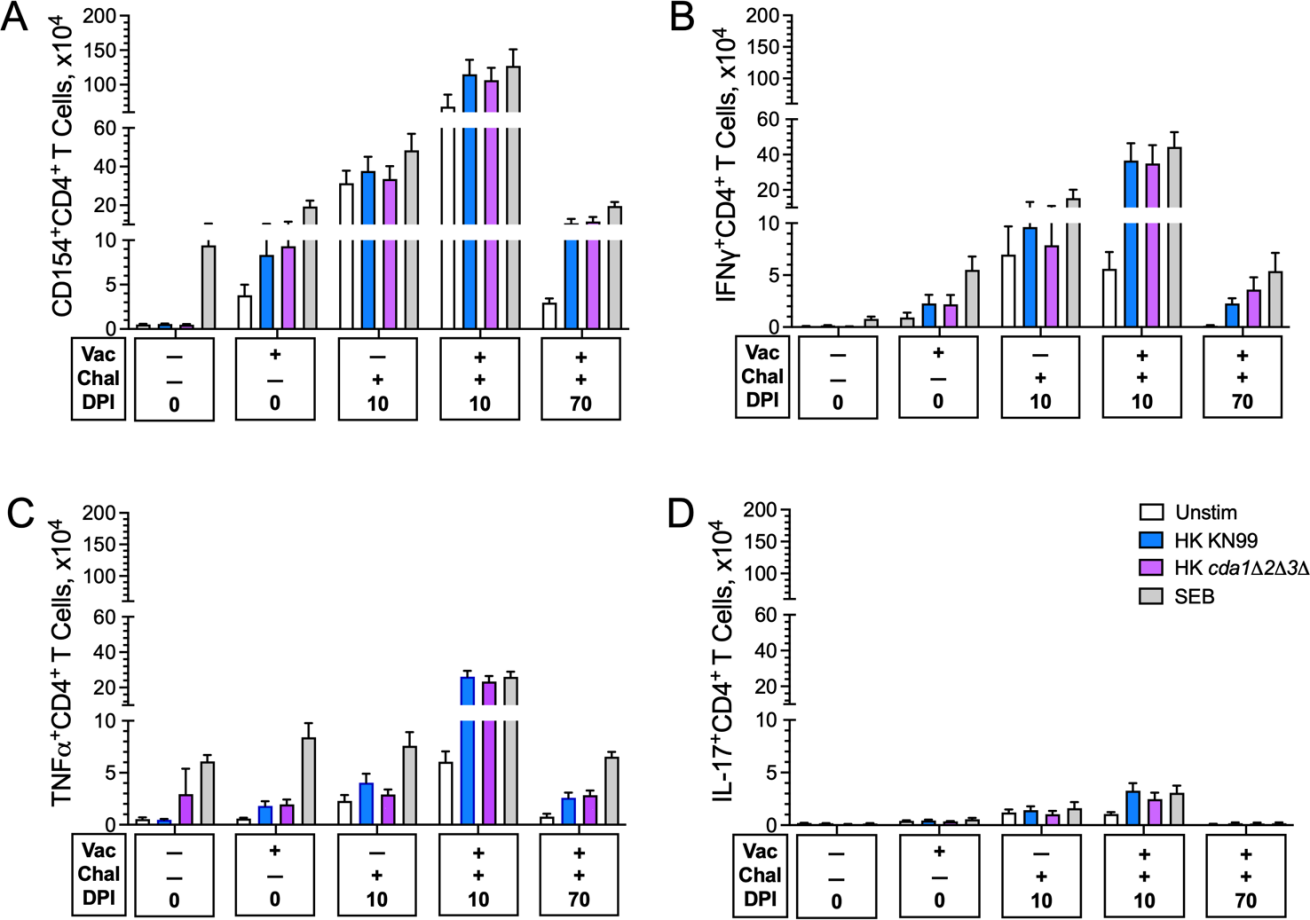
Ex vivo antigen-stimulated CD4^+^ T-cell activation and intracellular cytokine production following vaccination and/or infection. Lung leukocytes from the experiment shown in Fig. 6 were left unstimulated (Unstim) or stimulated with *Staphylococcus* enterotoxin B (SEB), HK KN99, or HK *cda1∆2∆3∆* for 18 h in complete media supplemented with 0.5-µg/mL amphotericin B. Brefeldin A was added during the last 4 h of culture. The cells were then stained and analyzed by polychromatic FACS. The numbers of CD4^+^ T cells expressing the activation marker CD154 (**A**) or producing the intracellular cytokines IFNγ (**B**), TNFα (**C**) and IL17A (**D**) following ex vivo stimulation are shown. Data are from two independent experiments, each with four to six mice/group. Data are presented as mean ± SEM. Statistical comparisons between groups are shown in Table S2. Chal, challenged with *C. neoformans* KN99; DPI, days post infection; Vac, vaccinated.

Next, we measured expression of the activation marker CD154 and the intracellular cytokines IFNγ, TNFα, and IL-17A by lung CD4^+^ T cells following ex vivo stimulation with HK *cda1∆2∆3∆* and HK KN99 (Fig. 7). These three intracellular cytokines were chosen for study due to their importance in host defenses against cryptococcosis (32–35) and the loss of protection phenotype seen in vaccinated mice deficient in IFNγ, IFNγR, TNFα, or IL-23p19 (Fig. 5). IL-23 is a key cytokine that promotes IL-17 production (37). Cells left unstimulated and stimulated with the superantigen *Staphylococcus* enterotoxin B (SEB served as negative and positive controls, respectively.

In mice that were vaccinated but not infected, both HK *cda1∆2∆3∆* and HK KN99 stimulated significant expression of CD154 (Fig. 7A), IFNγ (Fig. 7B), and TNFα (Fig. 7C), but not IL-17A (Fig. 7D) in the CD4^+^ T-cell lung population. By 10 DPI, all four of these subpopulations of CD4^+^ T cells expanded, with more robust expansion observed in mice that were both vaccinated and infected compared with infected alone. Contraction of CD154, IFNγ, TNFα, and IL-17A expressing CD4^+^ T cells was observed in vaccinated mice that survived 70 DPI. For all groups, the numbers of CD4^+^ T cells expressing these four markers were similar following stimulation with HK KN99 compared with HK *cda1∆2∆3∆*. Notably, modest increases over baseline numbers of CD154, IFNγ, TNFα, and IL-17A expressing CD4^+^ T cells were seen in groups that were vaccinated and/or infected but left unstimulated ex vivo. We postulate this is due, at least in part, to T-cell activation and stimulation from residual fungal antigens in the lungs.

### IFNγ production by ex vivo stimulated lung leukocytes following vaccination and/or infection

**Fig 8:**
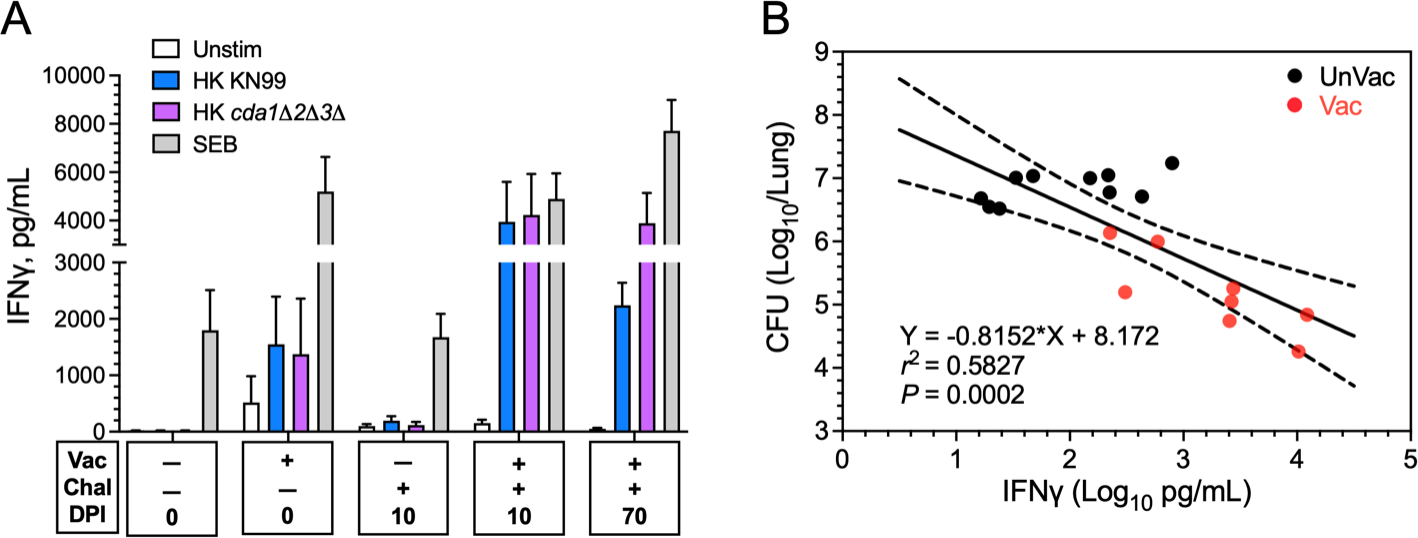
IFNγ production by ex vivo stimulated lung leukocytes following vaccination and/or infection. (**A**) Lung leukocytes were purified from vaccinated and/or infected mice and stimulated ex vivo for 18 h using the same mice and protocol as in Fig. 7. Supernatants were collected and analyzed for IFNγ by enzyme-linked immunosorbent assay. Data are means ± SEM. Statistical comparisons between groups are shown in Table S3. (**B**) Correlation between lung CFUs (see Fig. 6A) and lung leukocytes’ IFNγ levels following HK KN99 stimulation in individual mice at 10 DPI. Data were analyzed with simple linear regression and are presented with best-fit line and confidence bands. Pearson correlation was used for statistical analysis. Data are from two independent experiments, each with four to six mice/group. Chal, challenged with *C. neoformans* KN99; DPI, days post infection; UnVac, unvaccinated; Vac, vaccinated.

Many cell types in addition to CD4^+^ T cells make IFNγ (38). To get a broader picture of pulmonary IFNγ production in vaccinated and infected mouse lungs, we examined IFNγ production in the supernatants of lung cells from vaccinated and/or infected mice stimulated 18 h ex vivo with HK *cda1∆2∆3∆* and HK KN99 (Fig. 8A). As in Fig. 7, unstimulated and SEB-stimulated cells served as controls. Undetectable to low levels of IFNγ were found in supernatants from unstimulated lung cells. Neither HK *C. neoformans* preparation stimulated appreciable IFNγ in unvaccinated mice. In contrast, HK *cda1∆2∆3∆* and HK KN99 stimulated a vigorous IFNγ response in lung cells from vaccinated mice. Levels were even higher in mice that were vaccinated and infected. The high IFNγ levels persisted even at 70 DPI.

To examine further the contribution of IFNγ to vaccine-mediated protection, we looked at the correlation of lung CFUs and IFNγ levels in vaccinated and unvaccinated mice at 10 DPI. A highly significant inverse correlation between CFUs and IFNγ levels was found (Fig. 8B), lending further support for the critical contribution of IFNγ to vaccine-induced immunity.

## DISCUSSION

In humans and experimental mouse models, CD4^+^ T-cell immunity is required for protection against cryptococcosis (39). However, vaccines against cryptococcosis could work by eliciting protective responses from arm(s) of the immune system not in play during natural infection. Moreover, in situations where CD4^+^ T cells are deficient, immune plasticity could result in responses from cell types not required for protection in immunocompetent hosts. For example, an attenuated *Blastomyces dermatitidis* vaccine induced durable immunity in mice lacking CD4^+^ T cells by stimulating antigen-specific CD8^+^ T-cell responses (40). Thus, it is important to determine immunological mechanisms of protection as that will be informative for identifying the patient populations for which the vaccine is most likely to be efficacious. Therefore, we systematically examined the components of the immune system which mediate protection to the *cda1∆2∆3∆* vaccine.

A non-essential role for B cells was demonstrated as vaccine-mediated protection was retained in B cell-deficient muMT and JHD mice on the C57BL/6 and BALB/c backgrounds, respectively. These data suggest the *cda1∆2∆3∆* vaccine does not require antibody for protection. However, an ancillary contribution of antibody to protection could not be ruled out as there was a trend toward increased mortality in the vaccinated muMT mice compared to their wild-type counterparts.

*In vivo* and *in vitro* roles for CD8^+^ T cells in host cryptococcal defenses have been demonstrated (41–43). However, our data demonstrate that CD8^+^ T cells were not required for protection mediated by the *cda1∆2∆3∆* vaccine. First, the vaccine protected β2m mice, which are largely devoid of CD8^+^ T cells, from *C. neoformans* challenge as well as it did their wild-type counterparts. Second, mAb-mediated depletion of CD8^+^ T cells did not impact the survival of two strains of vaccinated mice.

In contrast, consistent with their paramount role in host defenses against cryptococcosis (3, 44), protection endowed by the *cda1∆2∆3∆* vaccine was completely lost in mice congenitally deficient in CD4^+^ T cells. Moreover, adoptive transfer of unfractionated T cells or the CD4^+^ T-cell subset from vaccinated mice conferred protection upon naïve mice. Depletion of CD4^+^ T cells using an anti-CD4 mAb abolished vaccine-mediated protection in mice if the depletion was performed at the time of vaccination. Importantly though, if CD4^+^ T cells were depleted after vaccination but prior to infection, then significant protection was retained. These results were consistent across three genetically distinct mouse strains: CBA/J, BALB/c, and C57BL/6, which have been described as highly resistant, moderately resistant, and susceptible, respectively, in mouse models of cryptococcosis (45). Thus, while CD4^+^ T cells are necessary to initiate a protective immune response, once the mice are immunized, effector cells are able to act in an apparently CD4^+^ T cell-independent manner to protect against fungal challenge. This plasticity has translational implications for persons with HIV/AIDS as it suggests vaccinating persons living with HIV while their CD4^+^ T-cell counts are relatively high could provide protection should their CD4^+^ T-cell counts then fall to the low levels (<100 cells/µL) associated with cryptococcosis (44). Studies in sub-Saharan Africa have found that the majority of patients presenting with AIDS-associated cryptococcosis had a history of being engaged in HIV care including receipt of anti-retroviral drugs (46, 47). Similarly, *cda1∆2∆3∆* vaccination could be offered to patients on transplant waiting lists in anticipation of their future need for immunosuppressive anti-rejection drugs (3). Protection against experimental cryptococcosis in the absence of CD4^+^ T cells has been described for other whole organism cryptococcal vaccines (15, 48). A caveat to the CD4^+^ T-cell depletion experiments is that resident memory lung T cells have been reported to be relatively resistant to depletion by anti-GK1.5 antibody (49).

Numerous murine and human studies have revealed IFNγ and TNFα are essential for host defenses against cryptococcosis (34, 39, 50–52). Indeed, the non-redundant contribution of these two cytokines in *cda1∆2∆3∆*-mediated protection was clearly demonstrated as we found a complete loss of protection in mice null for IFNγ, IFNγR, or TNFα. Our ex vivo studies identified Th1 cells as producers of these cytokines following *cda1∆2∆3∆* vaccination and *C. neoformans* infection. It has been postulated that polyfunctional T cells which make IFNγ and TNFα have enhanced effector function and correlate with protection following vaccination (53). The data in BALB/c mice are consistent with our previous studies in CBA/J mice demonstrating that following infection with *C. neoformans*, *cda1∆2∆3∆*-vaccinated mice had increases in pulmonary cytokines and chemokines often associated with Th1-type responses, including IFNγ and TNFα (11). Moreover, in the present studies, a significant inverse correlation was found between IFNγ levels and CFU in the lungs.

The heterodimeric cytokine IL-23 promotes differentiation and proliferation of IL-17-producing CD4^+^ cells (also known as Th17 cells) (37). Phenotypically, IL-23p19-deficient mice resemble mice deficient in IL-17 (54), although IL-23-independent production of IL-17 has been described (55). Protection mediated by the *cda1∆2∆3∆* vaccine was almost completely lost in mice genetically deficient in the IL-23p19 subunit, suggesting IL-17 is integral to vaccine efficacy. Interestingly, the Th1 response was considerably more robust compared with the Th17 response following ex vivo antigenic stimulation of vaccinated and/or challenged mice. This suggests that cell types other than CD4^+^ T cells are making IL-17 or that IL-23 has protective effects independent of IL-17 in our vaccine model. Multiple cell types are known to produce IFNγ, TNFα, and IL-17a (56–58), and the contribution of individual cell types to vaccine immunity merits further study. Recently, IFNγ and IL-17 production by γδ T cells was shown to contribute to protection afforded by the *C. neoformans* Δsgl1 vaccine (59). In addition to IL-23, the p19 subunit reportedly associates with the CD5 antigen-like (CD5L) protein; this p19/CD5L heterodimeric cytokine reportedly enhances the differentiation of granulocyte-macrophage colony-stimulating factor (GM-CSF)-secreting CD4^+^ T cells (60). Future studies will need to parse the role of p19 and to examine whether vaccine protection is lost in mice specifically deficient in IL-17 or GM-CSF.

In summary, our data shed light on the complex immunological mechanisms required for protection against cryptococcosis afforded by the *cda1∆2∆3∆* vaccine in mice. Several lines of evidence, when taken together, strongly suggest CD4^+^ T cells, particularly Th1 cells, play a central role. First, protection was lost in mice deficient in CD4^+^ T cells, either due to a genetic mutation or a mAb-mediated depletion. Second, protection could be restored by adoptive transfer of CD4^+^ T cells from vaccinated mice. Third, protection was lost in mice deficient in either IFNγ or IFNγR. Finally, mice vaccinated with *cda1∆2∆3∆* and then infected with *C. neoformans* had robust recruitment of Th1 cells to the lungs. Non-redundant roles for TNFα and IL-23p19 were also seen. Importantly though, once vaccinated, mice were protected from an otherwise lethal challenge even if their CD4^+^ T cells were depleted. These results suggest a translational path for the *cda1∆2∆3∆* vaccine; populations at high risk of cryptococcosis could be vaccinated while their CD4^+^ T-cell function is relatively intact.

## MATERIALS AND METHODS

### Reagents

Thermo Fisher Scientific (Pittsburgh, PA) was the source for reagents, except where noted. The buffer for flow cytometry staining was phosphate-buffered saline (PBS) supplemented with bovine serum albumin (0.5%). Complete medium for cell culture was RPMI 1640 containing 10% fetal bovine serum, 10-mM HEPES, 2-mM L-alanyl-L-glutamine (GlutaMAX), 100-U/mL penicillin, and 100-µg/mL streptomycin.

### Mouse strains

Table 1 lists the mouse strains used. CBA/J mice were purchased from Jackson Laboratories and housed in the ABSL2 facilities at Washington University. Other mouse strains were bred and housed in a specific pathogen-free environment in the animal facilities at the University of Massachusetts Chan Medical School. Unless indicated, mice of both sexes were used in approximately equal numbers. All transgenic mouse strains were periodically genotyped and/or phenotyped.

**TABLE 1:** Mouse strains^*a*^

Strain	Phenotype	Background	Source (reference)
C57BL/6	Wild type	C57BL/6	JAX #000664
BALB/c	Wild type	BALB/c	JAX #000651
CBA/J	Wild type	CBA/J	JAX #000656
NSG	Severely immunodeficient	NOD	JAX #005557 (21)
muMt	Mature B cell deficient	C57BL/6	JAX #002288 (61)
Jh (JHD)	Mature B cell deficient	BALB/c	Taconic #1147 (22)
TCRβ	α/β T-cell receptor deficient	C57BL/6	JAX #002118 (25)
β2m	CD8 T cell deficient	C57BL/6	JAX #002087 (26)
MHCII	CD4 T cell deficient	C57BL/6	JAX #003239 (62)
IFNγ	IFNγ deficient	C57BL/6	JAX #002286 (63)
IFNγR	IFNγ receptor 1 deficient	C57BL/6	JAX #003288 (64)
TNFα	TNFα deficient	C57BL/6	JAX #003008 (65)
IL-23p19	IL-23p19 deficient	C57BL/6	Genentech (54)

^*a*^ β2m, β2 microglobin; JAX, The Jackson Laboratory; muMT, mu (IgM) immunoglobin heavy chain; NSG, non-obese diabetic, severe combined immunodeficiency IL-2R common gamma-chain deficient; muMT, mu (IgM) immunoglobin heavy chain; TCRβ, T-cell receptor beta chain. Catalog numbers of the mouse strains are included with their sources and references, where relevant.

### Vaccinations and infections

Vaccinations and infections were with the avirulent *cda1∆2∆3∆* strain (18) and the highly virulent KN99α strain of *C. neoformans* (66), respectively. Studies with wild-type and genetically modified mice C57/BL6 and BALB/c were performed at UMass Chan Medical School. Stocks of the strains were maintained at −80°C as a mix of a yeast extract-peptone-dextrose (YPD) liquid culture shaken for 2 days at 30°C combined 1:1 (vol/vol) with 50% sterile glycerol. Prior to use in mouse studies, each stock was first grown on YPD agar medium at 30°C for 2–3 days and used as inoculum of liquid YPD cultures; plates were stored at 4°C for up to 30 days. For the first vaccination, *cda1∆2∆3∆* was shaken (225 rpm) in 25 mL of YPD in a 250-mL polycarbonate flask (CELLTREAT Scientific Products, Pepperell, MA) for 2 days at 30°C. For subsequent vaccinations, 14-mL polypropylene round-bottom tubes (Corning) and 4-mL YPD medium were used. Cells were collected by centrifugation for 5 min at 425 × *g*, washed once with PBS (equal in volume to the culture medium), and suspended in 10 mL (flask culture) or 4 mL (tube culture) of PBS. The suspended cells were then diluted 1:100 in PBS and counted using a TC20 automated cell counter (Bio-Rad Laboratories, Hercules CA). For the first vaccination with *cda1∆2∆3∆* (given to CBA/J, BALB/c, and C57BL/6 mice), the cell count was adjusted with PBS to 2 × 10^8^ cells/mL. For the second and third vaccinations (administered to C57BL/6 mice only), the cell count was adjusted to 2 × 10^7^ cells/mL. Mice were challenged with KN99 that had been cultured in 4-mL YPD medium and shaken for 18 h at 30°C. Cells were collected and washed, as above. The final concentration of KN99 depended on the strain of mice: for C57BL/6 mice, it was 2 × 10^5^ cells/mL; for BALB/c mice, it was 4 × 10^5^ cells/mL; and for CBA/J mice, it was 1 × 10^6^ cells/mL of PBS.

For the first vaccination with *cda1∆2∆3∆* and for infection with KN99, mice were individually anesthetized with isoflurane, USP (UMASS Department of Animal Medicine). Then 50 µL of the 2 × 10^8^ cells/mL suspension (described above) was administered by OT inoculation into the lungs (67). The second and third vaccinations of *cda1∆2∆3∆* were by SQ injection of 100 µL in the abdomen. KN99 was also introduced into the lungs by orotracheal inoculation of 50 µL.

The intranasal vaccinations and infections with CBA/J mice were performed at Washington University, as described (67, 68). Yeast cells (*cda1*∆*2*∆*3*∆ and KN99) were cultivated in YPD medium (50 mL of the medium in a 250-mL flask) and shaken at 300 rpm for 48 h at 30°C. Cells were collected by centrifugation at 3,000 × *g* for 10 min, and the pellet was washed twice with endotoxin-free PBS. PBS was added to the pellet to result in a final cell concentration of 2 × 10^8^ cells/mL (*cda1*∆*2*∆*3*∆ ) and 1 × 10^6^ cells/mL (KN99). To inoculate intranasally, approximately 5- to 6-week-old female CBA/J mice were anesthetized with an intraperitoneal injection (200 µL) of ketamine (8 mg/mL)/dexdomitor (0.05 mg/mL) mixture and then intranasally infected with 50 µL of the yeast cell suspension. After 10 min, the mice were administered with the reversal agent atipamezole (200 µL, 0.25 mg/mL) intraperitoneally. Reagents used for anesthesia were provided by the Washington University Division of Comparative Medicine Pharmacy.

For all infection experiments, mice were monitored daily; those showing signs of morbidity (including weight below 80% of pre-inoculation weight or extension of the cerebral portion of the cranium) were euthanized by CO_2_ asphyxiation and cervical dislocation. Survival studies were terminated at 70 DPI.

### CFUs

CFUs were determined for *cda1∆2∆3∆* and KN99 inocula administered to the lungs to corroborate their respective cell counts. Lung CFUs were measured following homogenization of lung lobes in 4-mL PBS containing 200-U/mL penicillin and 200-µg/mL streptomycin using an OMNI TH homogenizer with tip adaptor for 7 mm × 110 mm hard tissue probe (OMNI International, Kennesaw, GA). Dilutions were plated on Sabouraud dextrose agar (Remel) or YPD agar, and plates were incubated for 2–3 days at 30°C prior to counting. The total CFU per organ was computed, with a detection limit of 20 CFU per organ.

### CD4^+^ and CD8^+^ T-cell depletions

T cells were depleted from mice using mAb GK1.5 for CD4^+^ T cells (Bio X Cell, Lebanon, NH) and mAb 2.43 (Cell Culture Company, Minneapolis, MN) or YTS 169.4 (Bio X Cell) for CD8^+^ T cells. Each mAb was administered every 2 weeks by intraperitoneal (IP) injection of 200 µg in 100-µL PBS. Timelines for IP injections are depicted in Fig. 3.

### Transfer of T cells from vaccinated mice to naïve mice

BALB/c mice were vaccinated three times with *cda1∆2∆3*∆. The first vaccination was OT and the second and third vaccinations were SQ, as described above. Spleens and lymph nodes were collected from three to five mice euthanized 4 weeks after the third vaccination. Total T cells and CD4^+^ T cells were each purified by negative selection following the manufacturer’s instructions provided with the mouse Pan T Cell Isolation Kit and CD4^+^ T Cell Isolation Kit, respectively (Miltenyi Biotec, Bergisch Gladbach, Germany). Purified T cells were transferred to naïve BALB/c mice via the tail vein; each mouse was injected with 5 × 10^6^ cells in 100-µL PBS. Two days after transfer, the mice were infected with 2 × 10^4^ KN99 cells.

### Ex vivo stimulation and analysis of lung leukocytes

Lungs were collected and lung populations stimulated and analyzed as described (32), with minor modifications as noted. Briefly, lungs were collected after exsanguination by cardiac puncture and rinsed with PBS. Single-cell suspensions were prepared using the Miltenyi Lung Dissociation Kit (Miltenyi Biotec). Leukocytes were enriched and *C. neoformans* were depleted following centrifugation on a Percoll gradient (40%/67%; Cytiva, Uppsala, Sweden). Lung cells (4 × 10^5^ cells/well) were cultured for 18 h in 96-well flat-bottomed plates containing 200-µL complete media supplemented with 0.5-µg/mL amphotericin B. Stimuli included SEB (1 µg/ mL; Toxin Technology Inc., Sarasota, FL), HK KN99 (50 µg/mL), and HK *cda1∆2∆3∆* (50 µg/mL) for 18 h in complete media supplemented with amphotericin B (0.5 µg/mL). Brefeldin A (5 µg/mL; BioLegend, San Diego, CA) was added during the last 4 h of culture.

Plates were centrifuged at 825 × *g* for 5 min, and supernatants were collected for IFNγ ELISA (Mouse IFNγ DuoSet ELISA Kit; R&D Systems, Minneapolis, MN). Samples (50 µL) were assayed following the manufacturer’s instructions. The lower limit of detection of IFNγ was 10 pg/mL; values below the lower limit were assigned concentrations of 9 pg/mL. The leukocytes in the cell pellet were suspended and stained with the LIVE/DEAD Green Fixable Dead Cell Stain Kit (Invitrogen), followed by CD3-PE, CD4-PerCP/Cyanine5.5, and CD8-APC antibodies (BioLegend). Cells were then fixed and permeabilized using the Intracellular Fixation & Permeabilization Buffer Set, and FcRs were blocked with rat anti-mouse CD16/CD32 monoclonal antibody 2.4G2 (BD, Franklin Lakes, NJ), and then the cells were stained with antibodies CD154-PE/Cyanine7, IFNγ-BV650, IL-17A-BV510, and TNFα-APC/Cyanine7 (BioLegend). Antibody catalog numbers and their dilutions were the same as in Wang et al. (32). A five-laser Bio-Rad ZE5 flow cytometer (Bio-Rad) was used to acquire data. Data were analyzed using FlowJo version 10.10 software (BD). Gating was established using fluorescence minus one controls and isotype controls, as illustrated in Fig. S2.

### Statistics

GraphPad Prism version 10.1.2 (GraphPad Software, La Jolla, CA) was used for statistical analyses and drawing graphs. The Mantel-Cox log-rank test was used to assess significance when comparing Kaplan-Meier survival curves. Lung CFUs from the ex vivo studies had a non-parametric distribution; groups were compared using the Mann-Whitney test. Lung leukocytes, CD4^+^ T cells, and CD8^+^ T cells were normally distributed; groups were compared using one-way analysis of variance (ANOVA) with Tukey’s correction for multiple comparisons. Quantification of cytokine-producing CD4^+^ T-cell numbers or levels of IFNγ within supernatants post ex vivo stimulation was presented as mean ± SEM, and statistical comparisons were conducted using two-way ANOVA with either Tukey’s or Dunnett’s correction for multiple comparisons. For the correlation analysis between lung CFUs and IFNγ levels, the data underwent simple linear regression using Pearson correlation and were presented with a best-fit line and lines depicting 95% confidence intervals. A *P* value of <0.05 following corrections for multiple comparisons was considered significant.

## Data Availability

Data will be made fully available and without restriction.

## SUPPLEMENTAL MATERIAL

Supplemental Data mbio.01746-24-s0001.pdf Supplemental figures and tables.

ASM does not own the copyrights to Supplemental Material that may be linked to, or accessed through, an article. The authors have granted ASM a non-exclusive, world-wide license to publish the Supplemental Material files. Please contact the corresponding author directly for reuse.
