# Supplementary material for: Immunological correlates of protection mediated by a whole organism, Cryptococcus neoformans, vaccine deficient in chitosan
Source: mBio. 2024 Jul 9;15(8):e01746-24. doi: 10.1128/mbio.01746-24 (PMC11323574; doi:10.1128/mbio.01746-24)
Supplement: Supplemental Data — Supplemental figures and tables. [file mbio.01746-24-s0001.pdf]

**Supplementary Data files for Specht et al.**

**Supplementary Figure 1.**

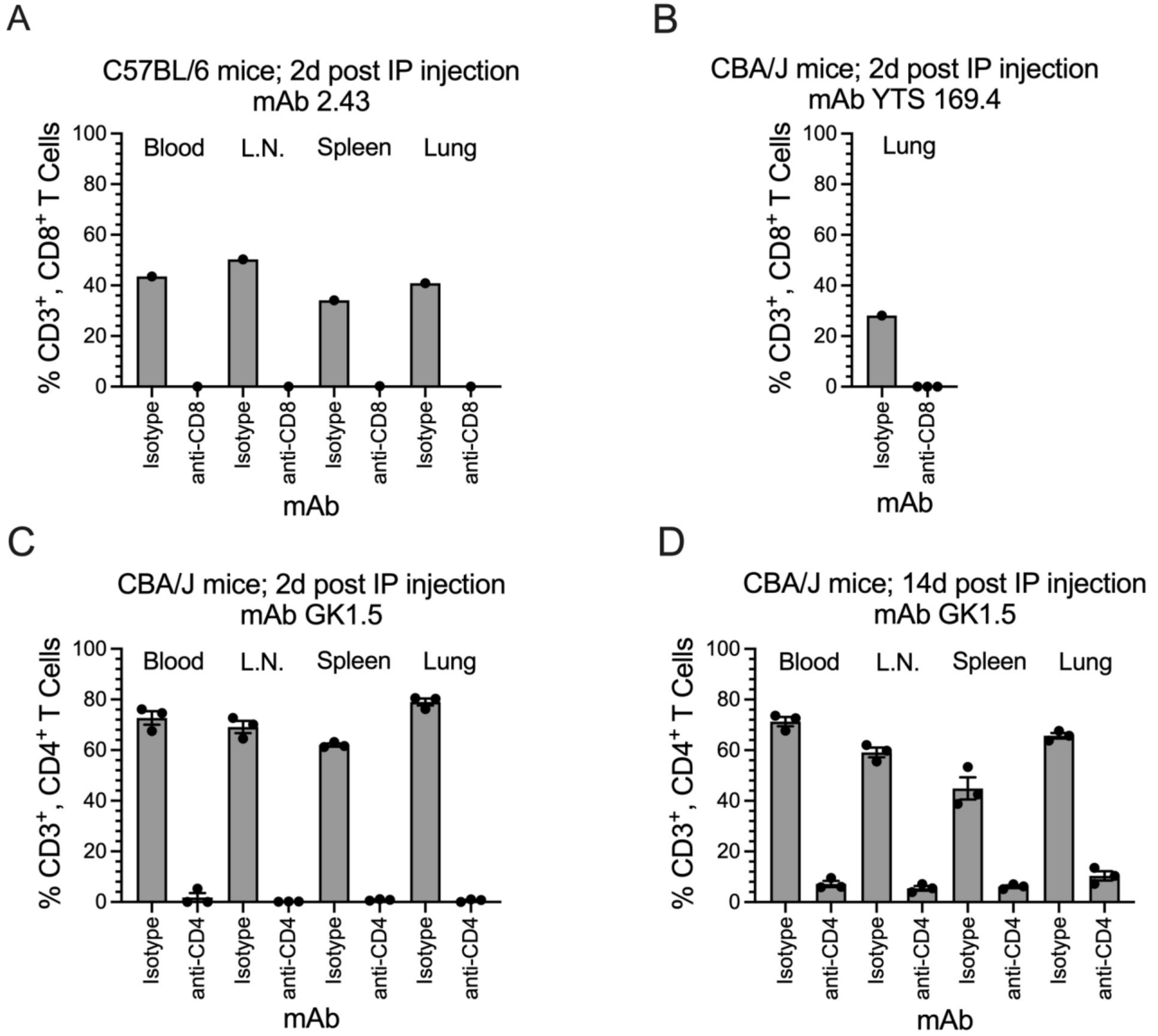


Please scroll down to the next page for the figure legend and methods for this figure.

**Supplementary Figure 1. Depletion of $CD8^+$ and $CD4^+$ T cells with monoclonal antibodies (mAbs)**. The indicated strain of naïve mice received 200 µg intraperitoneally (IP) of (**A**) the anti-CD8 mAb 2.43, (**B**) the anti-CD8 mAb YTS 169.4, or (**C, D**) the anti-CD4 mAb GK1.5. Control mice received the IgG2b isotype-matched mAb LTF-2. Mice were euthanized either 2d (**A, B, C**) or 14d (**D**) post injection following which the percent $CD8^+$ (**A, B**) or $CD4^+$ (**C, D**) T cells in the blood, inguinal lymph nodes (L.N.), spleens and lungs was determined. Closed circles represent individual mice. For n=3 mice, horizontal bars indicate means and error bars the SEM.

**Materials and Methods for Supplementary Figure 1**. Blood was collected by cardiac puncture with a 1 mL syringe and 26G needle filled with 50 uL of 0.5M EDTA. Spleen and lymph nodes were homogenized into single-cell suspensions by successively passing through 70 µm and 40 µm nylon strainers (BD Biosciences) using a syringe plunger. Lungs were enzymatically digested at 37°C for 30 min in RPMI 1640 containing 1 mg/mL of collagenase type IV (Sigma-Aldrich). The digested tissues were sequentially passed through sterile 70 µm and 40 µm pore nylon strainers. Erythrocytes were lysed by incubation in $NH_4Cl$ buffer (Sigma-Aldrich) for 3 min on ice, followed by addition of a twofold excess of PBS. Leukocytes were then collected by centrifugation, resuspended in sterile PBS, and stained using the LIVE/DEAD Fixable Blue Dead Cell Stain kit (Invitrogen) for 30 min at 4°C. Samples were washed and resuspended in FACS buffer (PBS, 0.1% bovine serum albumin (BSA), 0.02% $NaN_3$, 2 mM EDTA) and incubated for 5 min with CD16/CD32 (Fc Block; BD Biosciences). For flow cytometry, $10^6$ cells were incubated for 30 min at 4°C in the dark with fluorochrome-conjugated antibodies: anti-Mouse CD45, Clone: 30-F11, Pacific Blue (BioLegend), anti-Mouse CD3, Clone: 17A2, ACPcy7 (BioLegend), anti-Mouse CD4, Clone: RM4-4, FITC (BioLegend) and anti-Mouse CD8a, Clone: 53-6.7, BV650 (BD Biosciences). After three washes with FACS buffer, the cells were fixed in 2% ultrapure paraformaldehyde and sorted on a BD LSRFortessa X-20 flow cytometer. The data were analyzed with FlowJo v10 (TreeStar).

## Supplementary Figure 2.

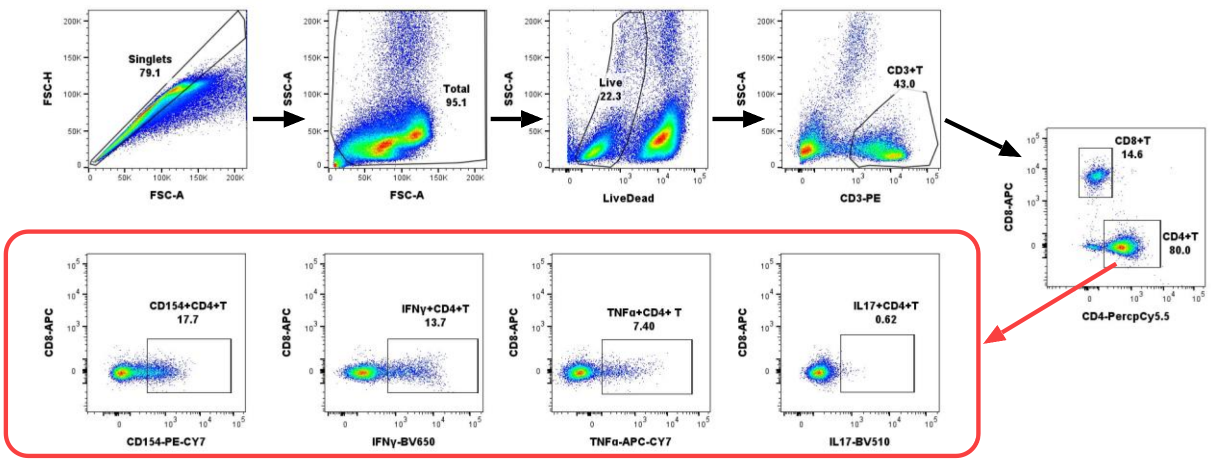


**Supplementary Figure 2. Flow cytometry gating strategy for analysis of T Cell activation and intracellular cytokine production in cultured lung leukocytes.** Singlet cells were gated based on forward scatter (FSC) height (FSC-H) versus area (FSC-A). Debris was excluded using a gating strategy based on FSC-A and side scatter area (SSC-A). Dead cells were further identified and excluded based on Live/Dead green staining. T cells were distinguished via CD3 staining, followed by selection of the $CD4^{+}CD8^{-}$ subset within the $CD3^{+}$ population. Subsequently, intracellular expression of CD154, IFNγ, TNFα, and IL-17A was analyzed within the live $CD3^{+}CD4^{+}CD8^{-}$ gated population, highlighted within a red box. Representative plots depict lung cells from mice administered a single dose of live *CnCda123* orotracheal vaccine, and analysis conducted 10 days post-challenge after ex vivo stimulation with heat-killed *KN99*.

**Supplementary Table 1. Statistics for group comparisons in Figure 6.**

| Comparison | Lung CFU | Leukocytes | $CD4^+$ T | $CD8^+$ T |
|---|---|---|---|---|
| | *P* | *P* | *P* | *P* |
| UnVac D0 vs. Vac D0 | - | >0.9999 | 0.9980 | 0.7634 |
| UnVac D0 vs. UnVac D10 | - | 0.0103 | 0.1210 | 0.9999 |
| UnVac D0 vs. Vac D10 | - | 0.0001 | 0.0005 | 0.9971 |
| UnVac D0 vs. Vac D70 | - | 0.9987 | 0.9997 | 0.9979 |
| Vac D0 vs. UnVac D10 | - | 0.0134 | 0.0746 | 0.6454 |
| Vac D0 vs. Vac D10 | - | 0.0002 | 0.0003 | 0.5683 |
| Vac D0 vs. Vac D70 | - | 0.9992 | >0.9999 | 0.6668 |
| UnVac D10 vs. Vac D10 | <0.0001 | 0.3925 | 0.1458 | 0.9996 |
| UnVac D10 vs. Vac D70 | 0.0007 | 0.0166 | 0.1580 | 0.9997 |
| Vac D10 vs. Vac D70 | 0.0016 | 0.0004 | 0.0015 | >0.9999 |

**Supplementary Table 1 legend.** As CFUs were not normally distributed, medians were compared by the Mann Whitney test. Means of leukocytes, CD4+ T and CD8+ T cell numbers were compared using One-way ANOVA with Tukey's correction for multiple comparison. P < 0.05 (shown after corrections for multiple comparisons) was considered significant different and indicated by red text. UnVac, unvaccinated. Vac, vaccinated.

**<u>Supplementary Table 2. Statistics for group comparisons in Figure 7.</u>**

| Comparison | | $CD154^{+}CD4^{+}$ T | $IFN\gamma^{+}CD4^{+}$ T | $TNF\alpha^{+}CD4^{+}$ T | $IL\text{-}17^{+}CD4^{+}$ T |
|---|---|---|---|---|---|
| | | ***P*** | ***P*** | ***P*** | ***P*** |
| UnVac D0 | Unstim vs. SEB | 0.8985 | 0.9992 | 0.0362 | 0.9999 |
| | Unstim vs. KN99 | >0.9999 | >0.9999 | >0.9999 | >0.9999 |
| | Unstim vs. *cda1Δ2Δ3Δ* | >0.9999 | >0.9999 | >0.9999 | 0.9977 |
| Vac D0 | Unstim vs. SEB | 0.6409 | 0.8398 | 0.0017 | 0.9845 |
| | Unstim vs. KN99 | 0.9842 | 0.9946 | 0.9018 | 0.9995 |
| | Unstim vs. *cda1Δ2Δ3Δ* | 0.9725 | 0.9954 | 0.8728 | 0.9997 |
| UnVac D10 | Unstim vs. SEB | 0.4373 | 0.3209 | 0.0138 | 0.7082 |
| | Unstim vs. KN99 | 0.9365 | 0.9373 | 0.6586 | 0.9330 |
| | Unstim vs. *cda1Δ2Δ3Δ* | 0.9970 | 0.9971 | 0.9760 | 0.9805 |
| Vac D10 | Unstim vs. SEB | 0.0004 | <0.0001 | <0.0001 | 0.0006 |
| | Unstim vs. KN99 | 0.0058 | <0.0001 | <0.0001 | 0.0002 |
| | Unstim vs. *cda1Δ2Δ3Δ* | 0.0299 | <0.0001 | <0.0001 | 0.0224 |
| Vac D70 | Unstim vs. SEB | 0.7621 | 0.8815 | 0.1251 | 0.9974 |
| | Unstim vs. KN99 | 0.9608 | 0.9885 | 0.8449 | 0.9975 |
| | Unstim vs. *cda1Δ2Δ3Δ* | 0.9472 | 0.9544 | 0.7926 | 0.9985 |
| Unstim | UnVac D0 vs. Vac D0 | 0.9996 | >0.9999 | >0.9999 | 0.9958 |
| | UnVac D0 vs. UnVac D10 | 0.2226 | 0.8072 | 0.9122 | 0.3090 |
| | UnVac D0 vs. Vac D10 | 0.0002 | 0.9179 | 0.0802 | 0.5193 |
| | UnVac D0 vs. Vac D70 | >0.9999 | >0.9999 | >0.9999 | 0.9998 |
| | Vac D0 vs. UnVac D10 | 0.3313 | 0.8702 | 0.9202 | 0.5509 |
| | Vac D0 vs. Vac D10 | 0.0005 | 0.9533 | 0.0848 | 0.7604 |
| | Vac D0 vs. Vac D70 | >0.9999 | >0.9999 | >0.9999 | 0.9878 |
| | UnVac D10 vs. Vac D10 | 0.0745 | 0.9994 | 0.3090 | 0.9985 |
| | UnVac D10 vs. Vac D70 | 0.4901 | 0.8925 | 0.9717 | 0.3840 |
| | Vac D10 vs. Vac D70 | 0.0043 | 0.9551 | 0.2307 | 0.5605 |
| SEB | UnVac D0 vs. Vac D0 | 0.9708 | 0.9573 | 0.8330 | 0.9538 |
| | UnVac D0 vs. UnVac D10 | 0.0657 | 0.1424 | 0.9483 | 0.0479 |
| | UnVac D0 vs. Vac D10 | <0.0001 | <0.0001 | <0.0001 | <0.0001 |
| | UnVac D0 vs. Vac D70 | 0.9825 | 0.9780 | 0.9998 | >0.9999 |
| | Vac D0 vs. UnVac D10 | 0.2801 | 0.5155 | 0.9944 | 0.2658 |
| | Vac D0 vs. Vac D10 | <0.0001 | <0.0001 | <0.0001 | 0.0001 |
| | Vac D0 vs. Vac D70 | >0.9999 | >0.9999 | 0.9516 | 0.9799 |
| | UnVac D10 vs. Vac D10 | <0.0001 | <0.0001 | <0.0001 | 0.0351 |
| | UnVac D10 vs. Vac D70 | 0.4748 | 0.6747 | 0.9929 | 0.1599 |
| | Vac D10 vs. Vac D70 | <0.0001 | <0.0001 | <0.0001 | 0.0002 |
| KN99 | UnVac D0 vs. Vac D0 | 0.9883 | 0.9980 | 0.9735 | 0.9887 |
| | UnVac D0 vs. UnVac D10 | 0.0904 | 0.5568 | 0.4038 | 0.1257 |
| | UnVac D0 vs. Vac D10 | <0.0001 | <0.0001 | <0.0001 | <0.0001 |

| | | | | | |
|---|---|---|---|---|---|
| | UnVac D0 vs. Vac D70 | 0.9777 | 0.9986 | 0.9059 | >0.9999 |
| | Vac D0 vs. UnVac D10 | 0.2717 | 0.7655 | 0.8084 | 0.3434 |
| | Vac D0 vs. Vac D10 | <0.0001 | <0.0001 | <0.0001 | <0.0001 |
| | Vac D0 vs. Vac D70 | >0.9999 | >0.9999 | 0.9978 | 0.9924 |
| | UnVac D10 vs. Vac D10 | <0.0001 | 0.0002 | <0.0001 | 0.0032 |
| | UnVac D10 vs. Vac D70 | 0.4652 | 0.8278 | 0.9671 | 0.2096 |
| | Vac D10 vs. Vac D70 | <0.0001 | <0.0001 | <0.0001 | <0.0001 |
| *cda1Δ2Δ3Δ* | UnVac D0 vs. Vac D0 | 0.9811 | 0.9978 | 0.9641 | 0.9917 |
| | UnVac D0 vs. UnVac D10 | 0.1660 | 0.7223 | 0.7605 | 0.3732 |
| | UnVac D0 vs. Vac D10 | <0.0001 | <0.0001 | <0.0001 | 0.0003 |
| | UnVac D0 vs. Vac D70 | 0.9687 | 0.9892 | 0.8693 | >0.9999 |
| | Vac D0 vs. UnVac D10 | 0.4659 | 0.8936 | 0.9902 | 0.6767 |
| | Vac D0 vs. Vac D10 | <0.0001 | <0.0001 | <0.0001 | 0.0017 |
| | Vac D0 vs. Vac D70 | >0.9999 | 0.9997 | 0.9962 | 0.9978 |
| | UnVac D10 vs. Vac D10 | <0.0001 | 0.0002 | <0.0001 | 0.0421 |
| | UnVac D10 vs. Vac D70 | 0.6607 | 0.9727 | >0.9999 | 0.5465 |
| | Vac D10 vs. Vac D70 | <0.0001 | 0.0003 | <0.0001 | 0.0019 |

**Supplementary Table 2 legend.** Means of cytokine-producing CD4+ T cells were compared using two-way ANOVA with Tukey's or Dunnett's correction for multiple comparison. $P < 0.05$ (shown after corrections for multiple comparisons) was considered significantly different and indicated by red text. UnVac, unvaccinated. Vac, vaccinated.

**Supplementary Table 3. Statistics for group comparison in Figure 8.**

| Comparison | | IFNγ level |
|---|---|---|
| | | *P* |
| Unvac D0 | Unstim vs. SEB | 0.3194 |
| | Unstim vs. KN99 | >0.9999 |
| | Unstim vs. *cda1Δ2Δ3Δ* | >0.9999 |
| Vac D0 | Unstim vs. SEB | 0.0004 |
| | Unstim vs. KN99 | 0.7218 |
| | Unstim vs. *cda1Δ2Δ3Δ* | 0.8170 |
| Unvac D10 | Unstim vs. SEB | 0.2760 |
| | Unstim vs. KN99 | 0.9994 |
| | Unstim vs. *cda1Δ2Δ3Δ* | >0.9999 |
| Vac D10 | Unstim vs. SEB | 0.0001 |
| | Unstim vs. KN99 | 0.0027 |
| | Unstim vs. *cda1Δ2Δ3Δ* | 0.0012 |
| Vac D70 | Unstim vs. SEB | <0.0001 |
| | Unstim vs. KN99 | 0.3269 |
| | Unstim vs. *cda1Δ2Δ3Δ* | 0.0318 |
| Unstim | UnVac D0 vs. Vac D0 | 0.9938 |
| | UnVac D0 vs. UnVac D10 | >0.9999 |
| | UnVac D0 vs. Vac D10 | >0.9999 |
| | UnVac D0 vs. Vac D70 | >0.9999 |
| | Vac D0 vs. UnVac D10 | 0.9956 |
| | Vac D0 vs. Vac D10 | 0.9978 |
| | Vac D0 vs. Vac D70 | 0.9974 |
| | UnVac D10 vs. Vac D10 | >0.9999 |
| | UnVac D10 vs. Vac D70 | >0.9999 |
| | Vac D10 vs. Vac D70 | >0.9999 |
| SEB | UnVac D0 vs. Vac D0 | 0.0416 |
| | UnVac D0 vs. UnVac D10 | >0.9999 |
| | UnVac D0 vs. Vac D10 | 0.0645 |
| | UnVac D0 vs. Vac D70 | 0.0005 |
| | Vac D0 vs. UnVac D10 | 0.0152 |
| | Vac D0 vs. Vac D10 | 0.9990 |
| | Vac D0 vs. Vac D70 | 0.3871 |
| | UnVac D10 vs. Vac D10 | 0.0245 |
| | UnVac D10 vs. Vac D70 | 0.0001 |
| | Vac D10 vs. Vac D70 | 0.2495 |
| KN99 | UnVac D0 vs. Vac D0 | 0.7056 |
| | UnVac D0 vs. UnVac D10 | 0.9999 |
| | UnVac D0 vs. Vac D10 | 0.0083 |

| | | |
|---|---|---|
| | UnVac D0 vs. Vac D70 | 0.4423 |
| | Vac D0 vs. UnVac D10 | 0.7366 |
| | Vac D0 vs. Vac D10 | 0.2416 |
| | Vac D0 vs. Vac D70 | 0.9845 |
| | UnVac D10 vs. Vac D10 | 0.0052 |
| | UnVac D10 vs. Vac D70 | 0.4591 |
| | Vac D10 vs. Vac D70 | 0.6703 |
| *cda1Δ2Δ3Δ* | UnVac D0 vs. Vac D0 | 0.7904 |
| | UnVac D0 vs. UnVac D10 | >0.9999 |
| | UnVac D0 vs. Vac D10 | 0.0037 |
| | UnVac D0 vs. Vac D70 | 0.0309 |
| | Vac D0 vs. UnVac D10 | 0.7878 |
| | Vac D0 vs. Vac D10 | 0.1057 |
| | Vac D0 vs. Vac D70 | 0.3152 |
| | UnVac D10 vs. Vac D10 | 0.0016 |
| | UnVac D10 vs. Vac D70 | 0.0218 |
| | Vac D10 vs. Vac D70 | 0.9988 |

**Supplementary Table 3 legend.** Mean IFNγ levels were compared using two-way ANOVA with Tukey's or Dunnett's correction for multiple comparison. *P* < 0.05 (shown after corrections for multiple comparisons) was considered significantly different and indicated by red text. UnVac, unvaccinated. Vac, vaccinated.
